# Gender differences in HIV-positive persons in use of cardiovascular disease-related interventions: D:A:D study

**DOI:** 10.7448/IAS.17.4.19516

**Published:** 2014-11-02

**Authors:** Camilla Ingrid Hatleberg, Lene Ryom, Wafaa El-Sadr, Amanda Mocroft, Peter Reiss, Stephan de Wit, Francois Dabis, Christian Pradier, Antonella d'Arminio Monforte, Martin Rickenbach, Matthew Law, Jens Lundgren, Caroline Sabin

**Affiliations:** 1 CHIP Department of Infectious Diseases, Rigshospitalet, University of Copenhagen, Copenhagen, Denmark; 2 Mailman School of Public Health, Columbia University, New York, USA; 3 Research Department of Infection and Population Health, University College London, London, UK; 4 Amsterdam Medical Center, University of Amsterdam, Stichting HIV Monitoring, Amsterdam, Netherlands; 5 CHU Saint-Pierre Hospital, Saint-Pierre Cohort, Brussels, Belgium; 6 ISPED, Centre Inserm U897, University of Bordeaux, Bordeaux, France; 7 Department of Public Health, Nice University Hospital, Nice, France; 8 Department of Health Sciences, San Paolo University Hospital, Infectious Diseases Unit, Milan, Italy; 9 Institute of Social and Preventive Medicine, University of Lausanne, Swiss Cohort Study, Lausanne, Switzerland; 10 The Kirby Institute, University of New South Wales, Sydney, Australia

## Abstract

**Introduction:**

There is a lack of data on potential gender differences in the use of interventions to prevent and treat cardiovascular disease (CVD) in HIV-positive individuals. We investigated whether such differences exist in the D:A:D study.

**Materials and Methods:**

Follow-up was from 01/02/99 until the earliest of death, 6 months after last visit or 01/02/13. Rates of initiation of lipid-lowering drugs (LLDs), angiotensin-converting enzyme inhibitors (ACEIs), anti-hypertensives and receipt of invasive cardiovascular procedures (ICPs; bypass, angioplasty, endarterectomy) were calculated in those without a myocardial infarction (MI) or stroke at baseline, overall and in groups known to be at higher CVD risk: (i) age >50, (ii) total cholesterol >6.2 mmol/l, (iii) triglyceride >2.3 mmol/l, (iv) hypertension, (v) previous MI, (vi) diabetes, or (vii) predicted 10-year CVD risk >10%. Poisson regression was used to assess whether rates of initiation were higher in men than women, after adjustment for these factors.

**Results:**

At enrolment, women (*n*=13,039; median (interquartile range) 34 (29–40) years) were younger than men (*n*=36,664, 39 (33–46) years, *p*=0.001), and were less likely to be current smokers (29% vs. 39%, *p*=0.0001), to have diabetes (2% vs. 3%, *p*=0.0001) or to have hypertension (7% vs. 11%, *p*=0.0001). Of 49,071 individuals without a MI/stroke at enrolment, 0.6% women vs. 2.1% men experienced a MI while 0.8% vs. 1.3% experienced a stroke. Overall, women received ICPs at a rate of 0.07/100 person-years (PYRS) compared to 0.29/100 PYRS in men. Similarly, the rates of initiation of LLDs (1.28 vs. 2.46), anti-hypertensives (1.11 vs. 1.38) and ACEIs (0.82 vs. 1.37) were all significantly lower in women than men (Table 1). As expected, initiation rates of each intervention were higher in the groups determined to be at moderate/high CVD risk; however, within each high-risk group, initiation rates of most interventions (with the exception of anti-hypertensives) were generally lower in women than men. These gender differences persisted after adjustment for potential confounders (Table 1).

**Conclusion:**

Use of most CVD interventions was lower among women than men in the D:A:D study. Our findings suggest that actions should be taken to ensure that both men and women are monitored for CVD and, if eligible, receive appropriate CVD interventions.

**Table 1:** Relative rate (RR) of receipt of each of the four interventions in women versus men, before and after adjustment for potential confounders (age, year, BMI, high cholesterol, high triglycerides, hypertension, previous MI, diabetes and moderate/high predicted 10-year Framingham CVD risk score)

	Before adjustment	After adjustment
Intervention	RR (95% CI); *p*-value	RR (95% CI); *p*-value
Lipid-lowering drugs	0.52 (0.49, 0.56); *p*=0.0001	0.80 (0.75, 0.86); *p*=0.0001
ACE inhibitors	0.60 (0.56, 0.65); *p*=0.0001	0.80 (0.74, 0.87); *p*=0.0001
Anti-hypertensives	0.81 (0.75, 0.86); *p*=0.0001	1.16 (1.07, 1.25); *p*=0.0001
ICPs	0.25 (0.20, 0.32); *p*=0.0001	0.49 (0.38, 0.63); *p*=0.0001

